# New Drugs, Appliances, and Things Medical

**Published:** 1892-01-02

**Authors:** 


### NEW DRUGS, APPLIANCES, AND THINGS MEDICAL.

[All preparations' appliances, novelties, etc., of which a notice is desired, should be sent for The Editor, to care of The Manager, 140, Strand, London, W.C.]

### CONDENSED MILK.

All experienced doctors, nurses, and mothers set great store by condensed milk. We do not belong to that class of persons who pull down one meritorious thing in order that they may more conspicuously exalt another; and, therefore, in our commendation of condensed milk we must not be understood to be speaking against fresh milk. Milk in any form, so long as it is pure and healthy, is an excellent food both for old and young. But now that we live in such very large towns and travel to the ends of the earth, where cows are not, it is an exceedingly difficult problem how to procure milk that is at one and the same time fresh and wholesome. As a matter of fact, the problem has not yet been solved. We cannot get fresh and healthy milk in our large towns, or on our journeys in distant countries, or on our military expeditions, in anything like sufficient quantities for health and comfort. The discoverers of the process of condensing milk in such a way as to preserve its cream and other constituents in a state of perfect wholesomeness have conferred an undoubtedly great boon upon civilised man, and especially upon the civilised child. It seems somewhat late in the day to be commending for use an article which is so universally known. But many of us still stick too much to the town cow and the town tap, for our own well-being. It is worth while to make a comparison between the best of condensed milk and ordinary fresh milk. We have no hesitation whatever in saying that condensed milk of the best brands is as much superior to a great deal of the fresh milk that is sold in every large town as the roast beef of old England is superior to the skinny products of the butcher's shop of an Italian village. Those who are familiar with Italian beef will appreciate the comparison. Condensed milk is for all practical purposes an admirable substitute for fresh milk of the very highest quality; whilst, as we have said, it is greatly superior to much of the fresh milk that is commonly in use. We have not space to indicate all the circumstances in which condensed milk in tins is available and convenient. But it is important to remember that one company in particular, the Anglo-Swiss Condensed Milk Co., of the Milkmaid Brand, provides us with at least four admirable preparations for every-day use. There is first the condensed milk pure and simple, which is of the highest excellence; there is next condensed milk with coffee; thirdly, condensed milk with cocoa, and fourthly, condensed milk with chocolate. We have given all these preparations a prolonged trial. They are wholesome, delicious, and exceedingly convenient. Probably, no well-appointed house is without them. The Anglo-Swiss Company do not give us Swiss milk only, but English milk, Aylesbury milk, condensed, and prepared after the Swiss fashion. There is, therefore, every possible guarantee of quality that a reasonable man could desire. It is almost superfluous to say that many infants, who cannot take ordinary cows' milk in any form, would die if it were not for condensed milk. Every considerable family will be richer in dietetic variety and better prepared to maintain health by keeping in store a good consignment of all the best brands and preparations of condensed milk.

We are provided with patterns of Dr. Lahmann's Reform cotton-wool underclothing. The material is elastic, unshrinkable, and soft as silk. Every variety of undergarment can be procured from the London Agency, 15, Fore Street, E.C. The garments are made of varying thickness, that all seasons may be provided for.

